# Repetitive Nonreentrant Ventriculoatrial Synchrony (RNRVAS)

**Published:** 2010-05-05

**Authors:** Johnson Francis

**Affiliations:** Malabar Institute of Medical Sciences, Calicut, Kerala, India

**Keywords:** pacemaker mediated tachycardia, repetitive nonreentrant ventriculoatrial synchrony

##  Introduction

Retrograde ventriculoatrial conduction in persons with implanted dual chamber pacemakers can cause two types of responses. One is the familiar pacemaker mediated endless loop tachycardia. Another less known response is the repititive non-rentrant ventriculoatrial synchrony (RNRVAS). Both conditions produce similar symptoms due to the loss of atrial booster function and the presence of the reverse atrial kick, which initiates neurocardiogenic responses.

## Pacemaker mediated endless loop tachycardia

In this form of tachycardia, the pacemaker actively participates in the tachycardia circuit. Retrograde conduction of a ventricular beat causes a P wave which is sensed by the the pacemaker circuit, triggering off the next ventricular stimulus at the end of the programmed AV delay. This cycle is repeated and results in an endless loop tachycardia at the programmed upper rate of the dual chamber pacemaker. This situation is more common in those who have been implanted a pacemaker for sinus node dysfunction, though retrograde conduction can be rarely intact in those with anterograde AV block. The trigger for the tachycardia is usually an appropriately timed ventricular premature beat. Occasionally it could be triggered by an atrial premature beat occurring within the post ventricular atrial refractory period. Pacemaker mediated endless loop tachycardia can be terminated by the application of a magnet and prevented by re-reprogramming the post ventricular atrial refractory period (PVARP). Interestingly, Barold et al [1] have reported an instance of induction of endless loop tachycardia by magnet application over a dual chamber pacemaker which persisted despite repeated removal and reapplication of the magnet. This odd behaviour of the pacing system occured only in the "magnet off" function and was due to the sensing of the magnet induced signals by the atrial circuit of the pacemaker. Magnet unresponsiveness of the endless loop tachycardia can also be because it gets converted into an RNRVAS [2]. The tachycardia reverts back to the pacemaker mediated endless loop tachycardia on removal of the magnet. If a programmer is not available, this arrhythmia may be terminated by chest wall stimulation which inhibits the ventricular channel of the dual chamber pacemaker.

## Repititive non-rentrant ventriculoatrial synchrony

RNRVAS [3] is less well known arrhythmia which can present with symptoms similar to that of the pacemaker mediated endless loop tachycardia. This rhythm also requires an intact ventriculoatrial conduction. Unlike the pacemaker mediated endless loop tachycardia, RNRVAS does not occur at the programmed upper rate interval. The retrograde activation of the atria occurs within the PVARP so that it is not sensed by the atrial circuit of the pacemaker. Though it is not sensed by the atrial circuit, it makes the atria refractory to the subsequent atrial paced output. The ventricular output is delivered by the device at the end of the programmed AV delay. The former is called functional undersensing while the latter is called functional loss of capture. Mulpuru et al [4] illustrates a case of RNRVAS in the interesting ECG section of the current issue of the journal. The electrogram shows two atrial signals for every ventricular signal.

A long AV interval as well as a fairly fast programmed lower rate favour the development of RNRVAS [3]. The faster rate can also be sensor driven in a DDDR pacemaker. RNRVAS can also trigger automatic mode switching because all atrial events are used to calculate the atrial rate.
